# Transferability in three dimensions (3D): applicability, theoretical engagement, and resonance

**DOI:** 10.1007/s10459-025-10439-2

**Published:** 2025-05-14

**Authors:** Megan E. L. Brown, Renée E. Stalmeijer, Bridget C. O’Brien

**Affiliations:** 1 School of Medicine, Newcastle University, Newcastle, UK; 2 School of Health Professions Education, Department of Educational Development and Research, Faculty of Health, Medicine and Life Sciences, Maastricht University, Maastricht, The Netherlands; 3 Department of Medicine, University of California, San Francisco, CA USA

**Keywords:** Transferability, Health professions education, Medical education, Qualitative, Methods, Quality

## Abstract

Though transferability is a multidimensional concept, our representations of transferability within Health Professions Education have largely focused on whether innovations or outcomes can be applied in different contexts. However, transferability has been variously described and used as a quality criterion through the history of qualitative research. To capture the complexity of this concept we propose a tri-dimensional conceptualisation of transferability, drawing on foundational qualitative research literature and our own experiences as qualitative researchers. We consider the origins of transferability within qualitative and Health Professions Education research, including its association with the concept of generalisability. We draw attention to shifts in debate, and the need for a clear conceptualisation of transferability as a concept. The three dimensions we propose, as part of our 3D transferability model, are: applicability; theoretical engagement; and resonance. By exploring these three dimensions, we highlight the importance of a critical, multifaceted approach to understanding and operationalising transferability. To promote practical application, we make suggestions regarding the use and discussion of each dimension of transferability. In doing so, we hope this exploration will be useful for scholars seeking to achieve broader impact of their evaluative work and empirical research.

## Introduction

Can you remember the first time you saw a 3D movie? Or the first time you wore a virtual reality headset? That first time you realised the recorded world didn’t need to be flat? The “real world” is not flat, of course, far from it, but our traditional mediums of capturing and presenting it often have been. The same can be said of Health Professions Education.

Traditionally, Health Professions Education has been conceptualised and studied in, arguably, two-dimensional ways. We have focused on cause and effect (Regehr, 2010), “neat” interventions (Dornan & Kelly, 2017), or on exploring complex phenomena purely descriptively, and in isolation (Kajamaa et al., 2019). We have made progress this way, but there is only so far that two-dimensional explanations and conceptualisations can take us in a three-dimensional world. Some concepts require more complex representation (Kelly et al., 2018). One such concept is the subject of this article – *transferability*. Though transferability is a multidimensional concept, in our experience, scholars often focus on only one dimension – namely, whether educational innovations or outcomes can be applied in different contexts.

Recently, we wrote an article on how to discuss transferability – a common quality criterion for qualitative research that concerns how others might learn from our findings, apply them in their own contexts, and build upon them in future research or practice – in qualitative health professions education research (Stalmeijer et al., 2024). We wanted to engage further with the concept of transferability because we see transferability as important, given the practical orientation of health professions education (HPE) research and the opportunities for highly localised research on practice to be broadly applicable when designed and conducted with rigour. We assumed the article would be relatively straightforward, but transferability turned out to be a slippery, multidimensional concept that has been variously described throughout the history of qualitative research. We realised that the concept was not flat and proposed three dimensions to offer a better representation: **applicability**,** theoretical engagement**,** and resonance**. We also recognised that engaging all three dimensions is not always necessary or appropriate when exploring and discussing transferability within a research project. Our previous article was a short overview. Now, in this longer article, we offer a more expansive, conceptual analysis of the origins, purpose, and praxis of transferability to detail the construction and recommended use of these three dimensions of transferability.

Transferability is commonly mentioned as one of four or five quality criteria for qualitative research, often under the umbrella of trustworthiness (Lincoln, 1995; Shenton, 2004, Stenfors 2020).The descriptions of transferability in these works generally focus on clear and comprehensive reporting on the research context so readers can determine relevance to other contexts. The other dimensions - credibility, dependability, confirmability, and, more recently, reflexivity - are often treated as distinct criteria. We suggest a more fluid view of these criteria, finding it difficult to disentangle them when thinking about quality in qualitative research. Thus, readers will likely see traces of these criteria in our discussion of the three dimensions of transferability.

To ground this discussion, we begin by tracing the origins of transferability within qualitative and health professions education research, considering the rising need for a clear conceptualisation of transferability as a concept within HPE. This exploration lays the foundation for our conceptualisation of transferability. Further, we make suggestions regarding the use and discussion of each dimension of transferability in qualitative HPE research, signalling the importance of thoughtful and critical use of the umbrella term “transferability”.

## The origins of transferability

The point of origin for transferability (at least as applied to qualitative research) seems to be generalisability – a key quality criterion for quantitative research.

However, what generalisability means within the context of qualitative research, and how it should be used, has been widely contested. While statistical-probabilistic generalisation (Polit & Beck, 2010) remains the most familiar form – associated with representativeness and extrapolation from samples to populations – other interpretations have emerged in response to discomfort with applying quantitative ideals to qualitative research (Firestone, 1993; Polit & Beck, 2010). Theoretical generalisability (relevance beyond a single study on the basis that findings extend or refine existing theory, (Varpio et al., 2021) and case-to-case translation (originating from programme evaluation literature (Firestone, 1993) and describing the process by which readers compare study contexts with their own) reflect attempts to reconcile this tension. However, even these forms of generalisability are not without limitation, often imposing implicit expectations of universality or applicability that do not always sit comfortably with the context-specific, interpretive nature of much qualitative inquiry. Terminology in this area has grown increasingly tangled, with scholars using terms like transferability, applicability, and translation interchangeably or inconsistently (Yilmaz, 2013; Varpio et al., 2021). This complexity is not unique to our field, but within health professions education, the historical weight of generalisability persists, and it remains often invoked as a shorthand for rigour and impact.

We are of the opinion that we are in need of a more conceptually robust definition of transferability. We believe qualitative research should not need to conform to quantitative language and standards (Lincoln & Guba, 2013; Yilmaz, 2013) and that, where quantitative concepts are used to critique qualitative research, this can inadvertently reduce rigour by forcing qualitative work into frameworks that overlook context, complexity, and meaning – the very foundations of qualitative inquiry. Therefore, transferability should be conceptualised on its own terms as a distinct, multidimensional construct that reflects the richness, complexity, and contextual sensitivity of qualitative inquiry, rather than as a type of generalisability.

However, there are points of alignment between the conceptualisations of generalisability we have outlined, and transferability. In particular, case-to-case translation facilitates the comparison of different educational contexts to evaluate the potential for adopting a programme or intervention. Whilst rooted in programme evaluation, case-to-case translation does underscore the importance of applying findings across settings, as transferability also does. This alignment highlights the spectrum on which these constructs exist – while some aspects of generalisability may overlap with transferability, transferability extends beyond comparison to encompass a deeper understanding of contextual dynamics and the multifaceted nature of HPE phenomena. It is related to generalisability, but a distinct, multidimensional entity.

Perhaps some of the confusion relating to transferability is because we have often assumed collective understanding of transferability in HPE, rather than making clear the gaps in our understanding. What our reading has taught us is that understandings of generalisability and transferability vary significantly, and that terminology in this area is complex. At times, complex terms are applied broadly to all qualitative approaches, regardless of more specific intentions/origins of the terms (e.g., of theoretical generalisability for studies with theory; and case-to-case translation for programme evaluation). Often, transferability is used as a “catch-all” concept when discussing the impact of a study’s findings, which can oversimplify the nuanced and diverse nature of qualitative research. This indiscriminate application of terms, without a clear understanding of their distinct meanings and contexts, makes it difficult to know how we can act on or use the findings of qualitative research across a variety of methodologies and approaches.

In this paper, we recognise this complex legacy and context but make the case for a more nuanced and conceptually robust approach to discussing how qualitative research findings might move beyond their immediate context within health professions education.

## Defining transferability

Transferability is widely used to describe the process of relating qualitative research findings to a context beyond that in which the study was conducted. The origin of the term is attributed to Lincoln (Coghlan & Brydon-Miller, 2014), who (alongside Guba) advocated for replacing the methodological concepts used in quantitative research with a unique set of terms for qualitative research – transferability was proposed as a substitute for generalisability (Lincoln & Guba, 1985), and as one of four qualitative research quality criteria (the others being confirmability, credibility, and dependability). Lincoln and Guba (1985) describe how transferability can be enhanced through “thick description”, or the provision of detail regarding the context and methodological processes of a study. They take the concept of “thick description” from Geertz (1973), but lose something in the translation of this idea, as in Geertz’s original work (1973) within anthropology, thick description also involves focusing on the layers of meaning within qualitative findings (Geertz, 2008). Interestingly, in Lincoln and Guba’s later work on qualitative research they replaced transferability with the concept of “cumulation” (Lincoln et al., 2011) – *“ways in which similar studies*,* carried out via qualitative methods with similar populations or in similar contexts*,* might be cumulated into meta-analyses” (p117)–* in new recognition that the concepts used within qualitative research should be unique to qualitative research, rather than a substitute for quantitative ideas. This is not yet a discussion we see echoed in our field, and there are possible issues with “cumulation” for interpretivist research, as the concept draws on quantitative understandings that synthesising large bodies of evidence through meta-analyses is, hierarchically, the highest quality of evidence.

Within health professions education, our understanding of transferability seems largely drawn from the original work of Lincoln and Guba, though there have of late, also been references (Varpio et al., 2021) to Firestone’s (1993) work on case-to-case translation. Ultimately, we would suggest that our use of transferability as a field is inconsistent. There is confusion about what terminology to use (Varpio et al., 2020) and often, transferability is not clearly discussed by articles. Where transferability is discussed, it is often poorly conceptualised (Varpio et al., 2020), or discussed only as a limitation of work without thought as to how, and why, it is a limitation (or whether it is a limitation at all) (Ross & Bibler Zaidi 2019). Where transferability is more clearly conceptualised, conceptualisations usually focus on the degree to which results can apply to other contexts, settings, or participants (Eva & Lingard, 2008; Tavakol & Sandars, 2014), which concords with both Lincoln and Guba’s work, and case-to-case translation. This application to other contexts is facilitated by the provision of “thick description”, about the context in which research was conducted, which allows readers to make a transferability judgement regarding whether findings apply to their own setting (Stenfors et al., 2020). However, we do not usually focus on explaining the causal relationships and meaning that might transcend the particulars of a situation, in the way that Geertz (1973) conceptualised thick description. Despite our inconsistent approach to conceptualising and discussing transferability, and, often, limited journal space to discuss transferability in appropriate depth, it continues to be used as a marker of high-quality research in our field – as LaDonna et al. (2021) suggest, transferable findings *“provoke thought*,* raise questions*,* and inform or change practice in settings beyond the research context”* (p.607).

As we traced the history of transferability as a concept and became aware of both Lincoln and Guba’s move away from the term, and our own experiences of unclear use of the term in health professions education, we were inspired to consider, in greater depth, how to conceptualise transferability in a way that inspires high-quality qualitative research and rigour. This led us to propose three dimensions: **applicability**, **theoretical engagement**, and **resonance**.

These dimensions can be understood as three ways of relating qualitative findings beyond their immediate context: practically, through applicability; conceptually, through theoretical engagement; and affectively, through resonance.

## Applicability, theoretical engagement, and resonance as three dimensions of transferability

Here, we discuss three dimensions of transferability, which synthesise our reading and interpretation of the various uses of transferability, and the needs it fulfils as a marker of quality in health professions education. These are: applicability, theoretical engagement, and resonance.

### The construction of applicability, theoretical engagement, and resonance

Before we move to discuss each dimension in-depth, and provide examples of their use, we felt it was important to offer some context on our creation of these dimensions. It is important to note that we do not believe there are only three dimensions of transferability. Rather, these three dimensions represent our current attempt as qualitative researchers and methodologists working in health professions education to provide a framework for researchers to consider how their work might engage with, and contribute to, conversations beyond their immediate context. We hope readers and methodologists will see this paper as an invitation to reflect on, adapt, and extend these ideas so that we may contribute to a living discussion about how we conceptualise and discuss transferability.

**Applicability** may feel most familiar, as it is the way in which we most commonly discuss transferability. Applicability connects with Lincoln and Guba’s conceptualisation of transferability through the idea that, in high-quality research, the findings of a study should be relevant and useful in other contexts. We have chosen to name this idea applicability, rather than transferability in its entirety, because it constitutes only one third, or one dimension, of our conceptualisation. Further, we believe that the term “applicability” better speaks to the active and intentional process of researchers choosing what to take from research and what to leave when considering their own, varied contexts.

Our second dimension is **theoretical engagement**. This aligns with theoretical generalisability in that it involves engagement with theory over the course of a research project. As previously referenced, the term “theoretical generalisability” (Varpio et al., 2021), though new to our field, captures this domain. We have chosen the language of “engagement”, rather than “generalisability”, for several reasons. First and foremost is to avoid flying too close to quantitative concepts, and to align ourselves firmly with qualitative approaches through the language of transferability. Secondly, we hope that the term “engagement” will be perceived as involving the continuous exploration, questioning, and refining of theoretical ideas as research unfolds – it is focused on the process of engaging with theory, whereas theoretical generalisability could be seen as outcome focussed. Our concern with encouraging an outcome-focused approach here is that it may inadvertently lead to researchers rushing their engagement with theory, or steer researchers towards identifying results that align with pre-existing theories, rather than allowing for the evolution of understanding through dynamic, ongoing dialogue between data and theory. Whilst we anticipate most qualitative research choosing to engage with this domain will engage explicitly with theory, we also anticipate this may take the character of conceptual engagement in some research. Where theories involve comprehensive systems of ideas that explain phenomena, concepts involve broader ideas, terms or categories that help in building theory (Brown et al., 2025). For example, community is a concept, whilst communities of practice is a theory.

**Resonance**, our third dimension, may feel less familiar than applicability and theoretical engagement, as it is not as extensively captured in the descriptions from our journey through generalisability and transferability. We have chosen to expand the conceptualisation of transferability to encompass resonance because we feel it captures the importance of emotional connection to research, and the importance of connection to experiences (personal, professional, and of others’ in the literature) which can drive deeper understanding, engagement, and, ultimately, action. We did not see this more affective domain captured by existing terms relating to applicability and theory. We adopted the idea of resonance into our conceptualisation of transferability based on our understanding of the importance of resonance within phenomenological research (Aagaard, 2018), and based on Ellingson’s (2009) work exploring resonance as a quality criteria for arts-based research.

Research projects may not consider all dimensions, though we anticipate that high-quality research will likely consider at least two. Each dimension serves as a standalone technique within the overarching concept of transferability, and so we suggest that authors specifically name which dimensions they have considered, and how, within any research outputs. There is no inherently “best” way to utilise these dimensions in a project – which a researcher selects to influence project design, delivery, and discussion will depend on the aims of the project, and methodological decisions. It is also important to note that these dimensions are not innate to any one project design or methodological choice, rather they are a collaborative choice between the research team, and the readers of a paper, who interpret what was done, and in which ways the paper meets these domains.

Below, we describe each dimension in detail, including what utilising the dimension throughout the lifespan of a project might look like in practice.

### Applicability

#### What is applicability?

Simply put, applicability is about whether the findings of one research study fit into different contexts (e.g., different settings, different learners) and can tell the reader of a paper something meaningful about their own world and experiences (Hammarberg et al., 2016). It is about relevance – looking beyond an individual study, to its implications across different contexts and groups, and what these implications mean for practice.

The use of applicability will vary depending on whether a project assumes a realist or relativist view of reality. For realist research, applicability will likely focus on sharing learning from educational innovations or research conducted in a particular context. The assumption of realist research is that there is an objective reality that exists independently of our understanding of it. The reason we are interested in applicability in realist research is usually to learn from educational innovations or research conducted in settings that are not our own. Though applicability means considering how context varies, the transfer of findings or implications from one setting to another implies that there is an underlying reality within which this transfer, or application is possible. The context that is considered as researchers write about their findings, and readers consider application, are differences in our understandings of reality, rather than actual reality itself.

Conversely, in interpretivist research (which typically views reality as relative, situated, or multiple), applicability’s focus is understanding the context in which the phenomenon was studied. There is no consistent underlying reality on which to apply understanding, so applicability is not so much about transplanting a ‘universal truth’ from one setting to another; it is about understanding the nuances, values, and interpretations that might shape the phenomenon in various contexts. Context interacts dynamically with the phenomenon being studied. In other words, while realist research aims for the transference of truths, interpretivist research seeks to provide a deep, contextualised understanding that readers can thoughtfully apply to their own unique circumstances. Therefore, the notion of applicability in interpretivist research tends to be more fluid, contingent on the complexities of human behaviour and the specificities of particular contexts. If using applicability within interpretivist research, this distinction in the conceptualisation and use of the term should be made clear.

#### How to consider applicability in a research project

Where appropriately used, the application of findings to a different context from which the study was completed requires thoughtful consideration, both on behalf of the research team, and the reader of a study. Researchers need to provide sufficiently appropriate and detailed information – aligned with Geertz’s original (1973) conceptualisation of “thick description” – so that readers can assess whether a study context is similar to their own, or what sort of considerations they may need to make in light of substantial differences. The term “thick description”, we feel, given its common use that has moved away from Geertz’s original conceptualisation, risks implying that a research team needs to include any and all information about their study to enhance applicability. This is not the case – the information needed to make a judgement regarding applicability may not need to be extensive – it depends on the study in question. Rather, we prefer the phrase “thoughtful description”, as this prompts researchers to consider: What do readers need to know about the context in which this study was conducted to help explain/make sense of these findings?

Building on our previous definition of context as concerning not only physical environments, but also the broader conditions under which behaviour or phenomena occur, we can extend this understanding further. Within applicability, considering context involves meaningful reflexivity on behalf of the research team, and a commitment to capturing the richness of participants’ experiences and the conditions in which those experiences unfold. While the term “descriptive” is sometimes used disparagingly in qualitative research, richly descriptive studies – those which go beyond surface-level reporting to offer layered, nuanced accounts of context, and meaning – can meaningfully support applicability. As Bates and Ellaway (2016) describe in their exploration of the “dark matter” of context, such work can cast light on the complex, often invisible forces that shape educational experiences. In these cases, depth and clarity of communicating context can allow readers to locate relevance within their own settings.

We wish to note here that there is also an important point to make regarding the relationship between thoughtful descriptions within applicability, and power. All too often, researchers working in the Global West (we are three such researchers) assume that their contexts (physical and sociocultural) are self-evident, or beyond the need for explanation. There is an inequitable power dynamic, where researchers working in the Global South are taxed with providing long explanations of how their context differs from the norm of the West. This is not only naive (clerkships, for example, differ in structure and organisation even within a country), but also reinforces inequality, particularly in light of often tight medical education journal wordcounts that make this task extremely difficult. We would stress that if researchers are using applicability, it is important for them to assume that their context is unfamiliar for any reader. When reading or reviewing research, reviewers and editors should also be wary of their own positionality, especially in relation to suggesting removing or adding contextual details from a paper. It is important to pause and consider how such recommendations could negatively impact readers in diverse settings.

There are several questions a research team can ask themselves to consider applicability within project design, data collection, and dissemination. These, we hope, might act as reflective prompts for a research team to consider during project design, data collection as they keep note on context throughout data collection (including participants’ sociodemographic characteristics), and when writing up findings as they decide the nature and detail of the content they need to provide in any manuscripts to allow readers to make an informed decision regarding applicability. We have outlined these questions in Table 1, below.

**Table 1:** Considerations to guide thoughtful description, with the aim of enhancing applicability

Nature of consideration	Ask yourselves:
Context	Have we effectively communicated the context, including not only physical context, but the social, historical, and situational factors that surround the behaviours or phenomena we are interested in? Are there any physical contexts, or concepts, that we have assumed our readers will understand? Why have we assumed these contexts or concepts are self-evident? Who might we be excluding if we do not provide an adequate description? While peer review can help surface assumptions and highlight areas where further clarification is needed, it can also reinforce field-specific norms, encouraging authors to exclude details considered ‘obvious’ within the community but inaccessible to broader audiences. How does our context relate to our findings and discussion? Looking at our results, are there any findings that require detail of physical context, or sociocultural context to be understood? Are these clearly highlighted, and discussed?
Interpretation	Have we paid careful attention to the voices and stories of our participants? In which ways have we lifted up participant voices? In which ways have we silenced them? Are participants’ stories connected to our interpretation of these stories (e.g., as embedded quotes)?
Meaning	Have we explained the significance or meaning of the behaviours or phenomena we have explored in the context in which they occur? How is this communicated to our reader? Are there layers of meaning in our analysis (i.e., are the findings descriptive, or analytical)? Have we considered and explained the cultural, social, and economic factors that influence the behaviour or phenomenon?
Reflexivity/Positionality	Have we critically reflected on our own role and backgrounds as researchers, considering whether we navigate inquiry from an insider’s perspective, sharing some experiences with participants, or from an outsider’s perspective, bringing external perspectives? How does our insider-outsider status influence how we approach analysis and interpretation? Have we provided a reflexivity statement that goes beyond a list of experiences in the team, and is a discussion of how these experiences influenced the research? In what ways might our backgrounds influence what we identify as important findings and recommendations? How might we discuss this in outputs clearly, and attend carefully to participants’ voices and experiences?
Richness	Does our description of our findings, and the meaning of these findings, feel “rich”*? *By “rich” we mean: does the data provide a detailed description of the topic of interest; does the data provide insight into the context in which the phenomenon of interest occurs/the topic is situated; does the data capture complexity; does the data feel authentic; and is there evidence of sustained and in-depth reflexivity. Different qualitative approaches invite different forms of richness – for example, phenomenological studies emphasise depth of understanding in relation to lived experience, while thematic or discourse analyses might highlight patterns, variation, and interpretive complexity. Generating richness is not only a property of the approach, but also of the researcher’s skill in asking thoughtful questions, creating space for participants’ stories, and working with data in ways that are sensitive to context, purpose, and audience.

These questions can inform research project decisions throughout the course of a project, if applicability is a key aim of the project. They can also guide the writing of the final project manuscript. Our observations suggest that applicability is most usually discussed in the methods of a research paper (where physical context is usually described) and in the discussion of a paper. Ideally, within a discussion, applicability will be considered, and key contextual considerations and their influence on the presented interpretation clearly discussed. Often, discussions of transferability are confined to the “limitations” section of a discussion, where a lack of applicability is lamented as a shortcoming of the project. We do not see the fact that the findings of a project may not be applicable to all settings as a limitation, rather as inherent to the context-bound nature of qualitative research, including realist qualitative research. Therefore, we recommend discussion of context and considerations for readers are woven throughout the discussion of a project where applicability is a domain of transferability that is utilised. Further, we believe it would be beneficial to signpost applicability as an aim of a project within the conceptual framework/introduction of a study. In our experience, applicability is often assumed but not articulated as an aim (e.g., the purpose of researching student experiences of a new curricula component may be to share learning on the topic so that other institutions can action these insights) – it would add clarity in terms of appreciating which domains of transferability a team have considered, if applicability is articulated as an aim, where appropriate.

#### Example of applicability in practice

In their study exploring trainees’ experience of simulation-based education within surgical training boot camps, Cleland et al. (2016), use ethnographic methods, including observations and interviews, to deeply explore the context surrounding the boot camps. Through thoughtful description of the specific historical context of surgical training, environmental considerations relating to the healthcare setting, cultural norms within the surgical community, and situational factors specific to the boot camp environment, the study provided rich insights into individuals’ learning, and in relation to the development and delivery of the boot camp.

By recognising the boot camp’s role in social and cultural processes, such as immersion in the culture of surgery, alongside individual cognitive learning, this study offers new directions for simulation-based education research and practice, through demonstrating the need for a more comprehensive understanding of educational interventions within medical education. It draws attention to the importance of moving beyond descriptions of outcomes, and of physical context, to social, cultural, and historical context. In doing so, and in connecting context directly to their findings and discussion, the researchers provided readers with essential information to assess the relevance of the study to their own settings. A medical school in a different country or cultural context could look at the study, understand the conditions under which the programme was successful, and consider applying similar strategies, tailoring them to their specific environment.

### Theoretical engagement

#### What is theoretical engagement?

Theoretical engagement describes the process of exploring, interpreting, applying, and discussing theory and/or theoretical concepts throughout the course of a research project with the aim to advance our understanding about a certain phenomenon. Theoretical engagement aids the researcher in making sense of the world and the ways in which it can be experienced (Varpio & Ellaway, 2021) by facilitating the de- or reconstruction and framing of complex phenomena (Hodges & Kuper, 2012; Samuel et al., 2020). This framing of phenomena on a more abstract and conceptual level enables the transferability of research findings beyond the context in which data were collected (Firestone). The process of “transfer” within theoretical engagement therefore occurs at a conceptual level rather than a literal, or factual level and involves critical, abstract thinking. More practically put, when developing a research project, a researcher will typically study the existing literature to identify relevant theories and/or concepts that might inform their approach to studying a complex phenomenon. This literature review will inform the process of generating a conceptual and theoretical framework in which the researcher explains how they will use theory and/or theoretical concepts as a lens to study the phenomenon they have identified (Maxwell, 2013).

The nature and level of theoretical engagement within a research project may vary. Sandelowski (1993) described how theoretical engagement can vary with regard to the source of theory, the centrality of theory use, and the temporal placement of theory within a study. The source of theory within a research project could be informed by existing, published theory. Alternatively, the research project may be the source through which theory is generated.For example, within the traditions of grounded theory and descriptive phenomenology, the research process often begins without prior theoretical committments, allowing constructus – or in some cases, theory – to arise as the product of empirical data (Sandelowski, 1993). In terms of the centrality of theory use, theory may be more peripheral to a study, only informing some aspects of its design or analysis., e.g. using theoretical concepts as sensitizing concepts during analysis or as a way to explain certain results. Theory could also inform all aspects of a research project from research question to methodological design, data analysis and discussion. The temporal placement of theory addresses at what point in time theory enters a research project; fully theory-informed, guiding all parts of the study design, or theory-informing inductive, in which theory is used to interpret data. See Table 2 for an overview of different approaches to theoretical engagement informed by the work of Varpio and colleagues (2020).

To some degree, the ontology that theoretical engagement implies depends on the nature of the theory with which the researcher engages (Brown & Dueñas, 2020). When a researcher uses a post-positivist theory or construct (e.g., Cognitive Load Theory) their theoretical engagement will likely assume a realist ontology. In this approach, the focus is on engaging with theory to identify and expand on mechanisms of how the world works, in ways that can be easily extrapolated to other contexts. If a researcher uses an interpretivist theory (e.g., Situated Learning) or construct, their theoretical engagement will likely assume an interpretivist ontology. Here, the aim is not to identify universal mechanisms, but to understand the subjective and context-specific experiences and meanings of individuals or groups within a particular sociocultural setting. Through a rich understanding of context, researchers can build understanding of the social and cultural factors influencing the experience of a phenomenon that can act as a useful basis for comparison, adaptation, and potential application of findings in similar contexts. There are provisos, of course, where researchers may use theories created in a particular paradigm, in a different paradigm to the theory’s origin – for example, Cognitive Load Theory in an interpretivist study. Here, researchers must be critical and reflective about how they integrate different theoretical perspectives, ensuring their research remains coherent, and considering their ontology clearly.

#### How to consider theoretical engagement in a research project

As mentioned, there are varied ways in which authors may choose to engage with theory within a research project. Communicating a research project’s approach to theoretical engagement and the ontology which informed choice of theory/theoretical concepts, are essential for fostering transferability through theoretical engagement. Identifying which approach to theoretical engagement fits your research project requires literature review, reflection and discussion. A researcher should ask themselves questions like ‘how does my positionality and theoretical orientation influence and contribute to my approach and interpretations?’, ‘which theory and concepts could inform my research, research question, and methodology?’. While conducting a research project and engaging in analysis, other questions are also likely to become relevant, such as ‘how do my findings align with my theoretical orientation?’, ‘what evidence am I providing to support this alignment?’ or, if one is aiming to build a theory or model, ‘what evidence am I providing to support this?’. Finally, a researcher should take care to reflect on the various ways in which their findings could relate to existing theoretical frameworks.

How and where the answers to these questions are communicated within a manuscript is dependent on the chosen approach to theoretical engagement. Language which may help to communicate a researcher’s approach to theoretical engagement is provided by Varpio et al. (2020) who offer a comprehensive overview of approaches to theoretical engagement. We have summarised these here (Table 2), and included suggestions on where to incorporate what authors may ask to guide their selection, but encourage readers to engage with Varpio et al.’s paper in full.

**Table 2:** Varpio et al.’s (2020) approaches to theoretical engagement within qualitative health professions education research

Approach to theoretical engagement	Description	Guiding questions for selection
Fully inductive theory development	Researcher develops a theory or theoretical model themselves, based solely on the data collected in their project.	Does your project aim to explore a new or under-researched area where existing theories might not apply?
Fully theory-informed inductive	Researcher uses an existing theory, theories, or constructs to guide project design, data collection, and analysis. Theory informs all aspects of the study. Theory can be modified or expanded based on the data collected.	Are there established theories that closely align with your research goals and can comprehensively guide your study design?
Theory-informing inductive	Researcher uses an existing theory or theories, to guide data analysis, where theory is used as an interpretative tool. Theory may be arrived at later within a project, and alter ongoing analysis. Theory can be modified or expanded based on the data collected.	Do you plan to use theory primarily as a lens for data analysis rather than as a basis for the entire research design?

Through engaging with theory in these diverse ways, and making clear one’s approach to theory, authors can enhance the transferability of their research. There is no singular “correct” way to engage with theory, nor a specific theory that is better than others in terms of transferability. Choosing an approach to theoretical engagement depends on several factors, including: project research question(s); existing literature in an area of study; and a researcher’s own worldview or values. Where there is rich existing literature on a topic, or on a related topic, it is likely best to build on this knowledge in a theory-informing approach, rather than attempt to create a new theory. For those interested in the organic experiences of participants, an inductive approach may be best.

#### Example of theoretical engagement in practice

Sawatsky et al. (2020), in their study “Coaching versus Competency to Facilitate Professional Identity Formation” can be said to engage with theory in a theory-informing, inductive way. Here, the authors engage with Goffman’s (1959; 2002) theory of impression management to analyse the tension between professional identity formation and assessment of competency within medical education. Goffman’s theory, which originally described how individuals present themselves to others in everyday social interactions, is applied to the context of medical training.

Further, the authors build on Goffman’s theory by suggesting that the system of medical assessment within postgraduate medical education in the United States encourages trainees to stage performances that emphasise competence, whilst hiding aspects of their practice or character which may lead to others believing they are weak, or incompetent. This adaptation of Goffman’s work to medical education led to a nuanced exploration and understanding of how traditional assessment methods may conflict with medicine’s self-stated goal of identity formation. By connecting Goffman’s theory with the practice of coaching, the authors use the theory to not only diagnose a problem, but to propose a solution. This example showcases thoughtful theoretical engagement that advances Goffman’s theory beyond its original context, and develops it into a framework for understanding and potentially transforming practice relating to professional identity formation within the field. This work could inform future interventions and policies which facilitate a more supportive educational environment, encouraging trainee authenticity.

### Resonance

#### What is resonance?

Resonance is a term with many meanings. In chemistry, resonance refers to the way electrons can be arranged in certain molecules (Reuter & Lüchow, 2021). In phonetics, resonance describes the richness of a person’s voice (Hawk, 2018). However, it is from the field of rhetoric that we find a definition of resonance most aligned with how we understand resonance in relation to transferability. In rhetoric, resonance describes the evocative power of a word, or text, and is used to refer to text which creates strong imagery, calls forth strong memories, or produces strong emotions in the reader of, or listener to, that text (Ettema, 2005). Resonant qualitative research is evocative and powerful. The observations and findings of resonant research are vivid, and stimulate emotional responses. Van Manen (1997) describes the “phenomenological nod”, where people reading a rich description nod their heads in agreement. Resonant qualitative research may invoke the phenomenological nod, or a deep sense of familiarity, of shared experiences between the stories captured by the text, and the reader.

It is important to note that for results to be resonant, readers do not have to share lived experience with the narratives presented. Rather, a paper should invite its reader to consider that experience through the depth of the data presented (i.e., the data are rich, and complexities are explored), the approach to analysis (to facilitate this depth), and the format of data (i.e., data are well-contextualised, connected, and accessibly presented).

The dimension of resonance assumes an interpretivist ontology – that reality is subjectively, and diversely experienced by individuals, and groups. Rather than attempting to make universal claims, resonance aims to present interpretations that readers with a variety of backgrounds and lived experiences can understand, relate to, or imagine, even if different from their own experiences. Simply put, if findings are resonant, they have skillfully embodied and interpreted the subjective realities of the research participants. Resonance is a quality criteria commonly-employed in arts-based research, where the value and impact of research are associated with the degree to which the artistic representations, narratives, and experiences of participants engage readers, and foster deep understanding of lived experience (Ellingson, 2009).

However, depending on resonance as a quality criteria can carry risks. Findings that feel deeply familiar to readers may be interpreted as “obvious” and therefore risk dismissal or rejection as lacking in novelty or contribution to a field’s literature base. Whilst this is a risk, especially for those navigating academic publishing, the value of confirming and consolidating complex phenomena, especially across contexts, should not be underestimated. There are also instances within academia where something may be widely felt and understood, but under-articulated, especially empirically. In these cases, research that gives form and language to shared but previously unspoken experiences or assumptions can make a critical contribution. To us, familiarity should not undermine contribution – rather, resonance’s role is to reinforce collective understanding, build shared language, and inspire action. We caution against treating novelty as the sole marker of value for qualitative health professions education research, and encourage future conceptual work to explore the connections between novelty and resonance in greater depth.

#### How to consider resonance in a research project

To consider resonance in their own work, researchers must first reflect on how they experience resonance – what it feels like. We anticipate that each of us will sense resonance in distinct ways – we may be captivated by vivid findings, experience an emotional, or visceral resonance, or find that reading a paper causes us to recall or imagine experiences of our own. Given that resonance depends on our own lived experiences, the concepts of resonance and reflexivity are intimately intertwined. It is important to design a research project aiming for resonance with reflexivity at the forefront of individual and team thinking. It is also important to reflect on resonance as a reader of research, and as a reviewer of academic research. We suggest that the following questions, modelled on Absolon (2022), may prove useful for considering your positionality in relation to resonance, and enable more inclusive project design and delivery: Table 3.

**Table 3:** Reflexive prompts as a starting point to considering the intersection between reflexivity and resonance within qualitative research for researchers, readers, and reviewers

Nature of consideration	Ask yourselves:
Individual experience	What aspects of your unique lived experience might influence the resonance of your research findings?
	What is less likely to be resonant for your target audience based on your perception of their experiences?
Understanding others’ experiences	How do you anticipate your experiences will shape the way you present and interpret the experiences of others in your research?
Social and cultural influences	How have factors like whiteness, colonialism, classism, sexism, ableism, and hetero/cisnormativity influenced your research approach and findings?
Research context	How might the context in which your research was conducted influence its resonance with your target audience of readers?
Patterns of resonance	Are there patterns in the feedback you receive from readers regarding what resonates within your work? Why/why not?

Power dynamics play a critical role in shaping resonance in research – dismissing research on the basis of a lack of resonance could be rooted in bias, and discrimination, rather than a lack of attention to resonance by the author. We must approach resonance in a reflexive and critical way throughout the entire duration of a project. In addition to enhancing individual and team reflexivity, there are other important considerations that may facilitate the resonance of a research project. The introduction of a paper plays an important role in establishing the importance of a project, and signposting to whom the paper is likely to be particularly important (and, hopefully, resonant). Given that findings must be accessible, clear descriptions of data collection and analysis methods, as well as a description of the way in which findings are presented (e.g., what labels for quotes mean) are important. Resonant findings are likely to pay close attention to context, and offer detail on the complexity and layers of meaning within a data set. Within the discussion section of a paper, projects aiming for resonance are likely to relate the findings of the project to wider literature to discuss the impact of the findings, and actions moving forwards.

#### Example of resonance in practice

For an example of drawing on resonance as a domain of transferability within qualitative research, we turn to the method of poetic inquiry. This is a newer, arts-based research method within health professions education (Brown et al., 2021), which involves the creation of poems from participant data, research literature, or self-reflective insights.

Brown et al. created poems from in-depth, phenomenological interviews with new doctors who had recently made the transition from medical school to clinical practice. The study’s use of poetic inquiry not only adds depth to the understanding of this critical career transition, but also provides a unique method through which readers can engage with the subject matter. The poems presented in the paper act as both data and inspiration, fostering multi-layered interpretations that enable readers of diverse backgrounds to connect or resonate with participants’ experiences. Through close attention to language, rhythm, form, and the lived experiences of participants, poetic inquiry can lead to resonant research by conveying the emotional weight of experiences, and through measures to enhance the strength of participants’ own voices within the data.

## What does this mean for qualitative research?

With the aim of offering a more thoughtful vocabulary for addressing the transferability of qualitative research, we have constructed three new dimensions of transferability – 3D transferability. Though (we hope) these domains better acknowledge qualitative-specific, diverse quality criteria, we would also caution that these domains are not the only marker of quality within qualitative research. Next to credibility, dependability, and confirmability, alternative approaches within interpretivist qualitative research, such as arts-based approaches, may draw on different criteria. For example, the aesthetics of a piece of work, or the interest of the work.

Our three dimensions also reflect and intersect with more traditional markers of qualitative quality. Applicability aligns most closely with what might be considered “classic” descriptions of transferability, while also encouraging researcher reflexivity. Theoretical engagement ties loosely to credibility, as it foregrounds clarity and coherence in the use of theory across a study. Resonance carries traces of confirmability, attending to the connection between data and findings—but it also moves beyond that, inviting emotional and intellectual recognition from readers. In this way, our 3D model supports a more nuanced and practice-oriented understanding of how transferability might be articulated within different qualitative traditions.

Whilst we hope these domains will help qualitative researchers in our field consider the external impact of their work, or how their work engages in a conversation with other researchers and practitioners, we also encourage readers to think critically about 3D use, and be judicious about which domains they choose to engage with, and when. It is not always appropriate to draw on all three domains; this is acceptable and expected. What is important is, in (ironically) quantitative terms, to “show your work” – why have you chosen to engage with a certain domain, how have you done this, have you answered your research question(s), what have you achieved? However, we recognise that journal word counts can constrain the extent to which qualitative researchers are able to make these decisions visible. Transparency around transferability choices often sits in tension with word limits. We encourage editors and reviewers in our field to be generous in their expectations and to recognise that demonstrating the reasoning behind dimension selection and method choices is part of robust qualitative reporting, and most often cannot be compressed into brief, formulaic statements. Word counts that enable depth are an important part of supporting quality qualitative research.

We have presented a conceptualisation of transferability that we feel represents current thinking and practice within health professions education. While we have proposed three dimensions—applicability, theoretical engagement, and resonance—as a framework for considering transferability, we do not claim these are the only possible dimensions. Rather, we see our 3D model as a starting point, as a way of opening a conversation about how we articulate and evaluate transferability for qualitative research. As our field’s research practices continue to change, we anticipate that new dimensions may become apparent, or existing ones may shift. It is our hope that researchers, educators, and methodologists will engage critically with our framework – adapting or challenging it – so that we can, collectively, advance a more nuanced and context-sensitive understanding of transferability. For now, we see transferability in three dimensions… but the field decides what comes next.

## Data Availability

No datasets were generated or analysed during the current study.
